# Sacral osteotomy combined with triangular osteosynthesis in the treatment of malunion and nonunion of vertically displaced pelvic fractures

**DOI:** 10.1186/s13018-022-03296-x

**Published:** 2022-09-05

**Authors:** Yangxing Luo, Li He, Yue Li, Jie Xie, Song Gong, Qian Zhang, Enzhi Yin, Meiqi Gu, Chengla Yi

**Affiliations:** Department of Traumatic Surgery, Tongji Hospital, Tongji Medical College, Huazhong University of Science and Technology, Jie Fang Avenue 1095, Wuhan, 430030 China

**Keywords:** Sacral osteotomy, Triangular osteosynthesis, Pelvic fractures, Malunion, Nonunion

## Abstract

**Background:**

Malunion and nonunion of vertically displaced pelvic fractures result in lower limb length discrepancies, claudication, and pain. There have been few previous reports of this type of corrective surgery for these old pelvic fractures. We present a surgical technique of sacral osteotomy combined with triangular osteosynthesis in the treatment of malunion and nonunion of vertically displaced pelvic fractures and report on its short-term clinical results.

**Methods:**

We retrospectively reviewed nine patients (five males and four females) with malunion or nonunion of vertically displaced pelvic fractures treated with sacral osteotomy and triangular osteosynthesis from April 2015 to January 2020. The age ranged from 14 to 45 years (average, 30.7 years). The time from injury to deformity correction surgery ranged from 3 months to 5 years (average, 12.8 months). The vertical displacement of a unilateral hemipelvis was 3.0–4.5 cm (average, 3.80 cm). According to AO/OTA classification at the initial fracture, there are eight cases in type C1.3 and one case in type C3.3. Sacral osteotomy and triangular osteosynthesis were used in all nine patients. The degree of unilateral hemipelvic reduction was assessed postoperatively based on measurements from the anteroposterior (AP) X-ray. Majeed score and pain visual analog scale (VAS) were used to assess the therapeutic effect of the patients during follow-up.

**Results:**

In all nine patients, postoperative AP X-ray showed correction displacement of 1.7–3.9 cm (average, 3.20 cm). All the patients were followed up for 6–36 months (average, 12.7 months). At the last follow-up, the Majeed score of pelvic fracture increased from an average of 53.9 points (30–84 points) preoperatively to 87.0 points (72–94 points), and the VAS score for pain decreased from an average of 6.0 points (4–8 points) preoperatively to 1.2 points (0–3 points). None had complications like infection, implant broken, screw loosening, iatrogenic nerve, and blood vessel injury.

**Conclusion:**

Sacral osteotomy combined with triangular osteosynthesis for the treatment of pelvic malunion and nonunion caused by sacral fractures can correct significantly vertical displacement of a unilateral pelvis, prolong limb length, and reconstruct the stability of a pelvic ring, achieving good clinical results.

## Introduction

Sacral fractures with vertical displacement are one of the type C pelvic fractures, which are often caused by high-energy injuries and are frequently associated with multiple injuries [1]. In the early stage, the treatment of life-threatening bleeding and associated injuries is predominant, and the reduction of pelvic fractures is absent. When the patients are stable both physiologically and hemodynamically, the fresh fractures turn into old. Due to the complex and high risk of surgery of old pelvic fractures, sophisticated surgical techniques and related equipment are required. Insufficient techniques and lack of related equipment led to pelvic fractures do not been surgical reduction and rigid internal fixation, which eventually cause malunion or nonunion [2, 3]. The malunion or nonunion with the significant vertical displacement of a unilateral pelvis results in the unbalanced transmission of axial forces from the spine to the lower limbs, causing lower limb length discrepancies, gait instability, posture problems (sitting and standing imbalance), pain in the posterior and anterior pelvic ring, and aesthetic defects [3–6].

In order to restore the normal morphology of the pelvis, osteotomy of anterior and posterior pelvic rings is often required to correct the vertical displacement of a unilateral pelvis [3]. Reduction and fixation are also indispensable after osteotomy. Kach and Trentz et al. [7] reported lumbopelvic distraction osteosynthesis (LPDO) to treat the vertical displacement of posterior pelvic ring injuries. It used a pedicle fixation system to distract longitudinally between L4, L5, and the iliac crest, and especially apply to sacral fractures with significant vertical displacement. Combined with transverse fixation (sacroiliac screw or transiliac/transsacral plate), it forms triangular osteosynthesis which has been proved biomechanically to be the most stable fixation of the posterior pelvic ring [2]. This construct provides multiplanar stability.

Due to the above reasons, we used sacral osteotomy combined with triangular osteosynthesis to treated nine patients with pelvic malunion and nonunion originating from sacral fractures from April 2015 to January 2020. In this study, we retrospectively reviewed these data to (1) present the surgical technique of sacral osteotomy and (2) analyze the clinical effect of this treatment from the clinical and radiological aspects.

## Patients and materials

### Demographic data

This retrospective study was approved by the local ethics committee, and written informed consent was obtained from each participant. Between April 2015 and January 2020, 9 patients with nonunion and malunion of vertically displaced pelvic fractures treated with sacral osteotomy and triangular osteosynthesis were included in this study for assessment.

The inclusion criteria were as follows: posterior pelvic ring fractures originating from sacral fractures; more than 2 cm of unilateral vertical pelvic displacement on anteroposterior (AP) X-ray examination; lumbosacral pain excluding other causes, pelvic nonunion or malunion; and age under 60 years.

The exclusion criteria were as follows: severe internal medical diseases resulting in an inability to tolerate surgery; severe mental illness precluding cooperation with surgical management; incomplete medical records; and a follow-up time less than 6 months.

### Preoperative preparation

The patient's neurological function was assessed for associated loss of lower limb motor function and skin sensation before the operation. Activities of daily living were scored according to the Majeed scoring system in terms of five aspects: pain, standing, sitting, sexual intercourse, and work performance [8, 9]. The degree of pain was scored according to the visual analog scale (VAS). Laboratory indexes were detected to rule out infection, including the white blood cell (WBC) count, erythrocyte sedimentation rate (ESR), and C-reactive protein (CRP) level. Imaging data were used to assess the degree of fracture displacement and callus formation, including AP, inlet, and outlet X-ray and three-dimensional computed tomography (3D-CT) images. The unilateral vertical pelvic displacement was determined by drawing two horizontal lines across both acetabular apexes perpendicular to the spinous processes on the AP view of the pelvis and measuring the perpendicular distance between the two horizontal lines. Fasting, water deprivation, skin preparation, and indwelling catheterization were required before surgery. An enema was performed the night before and the morning of surgery. Sufficient plasma and concentrated red blood cells were prepared, along with an autologous blood transfusion device before surgery.

### Operative procedure

**Fig. 1:**
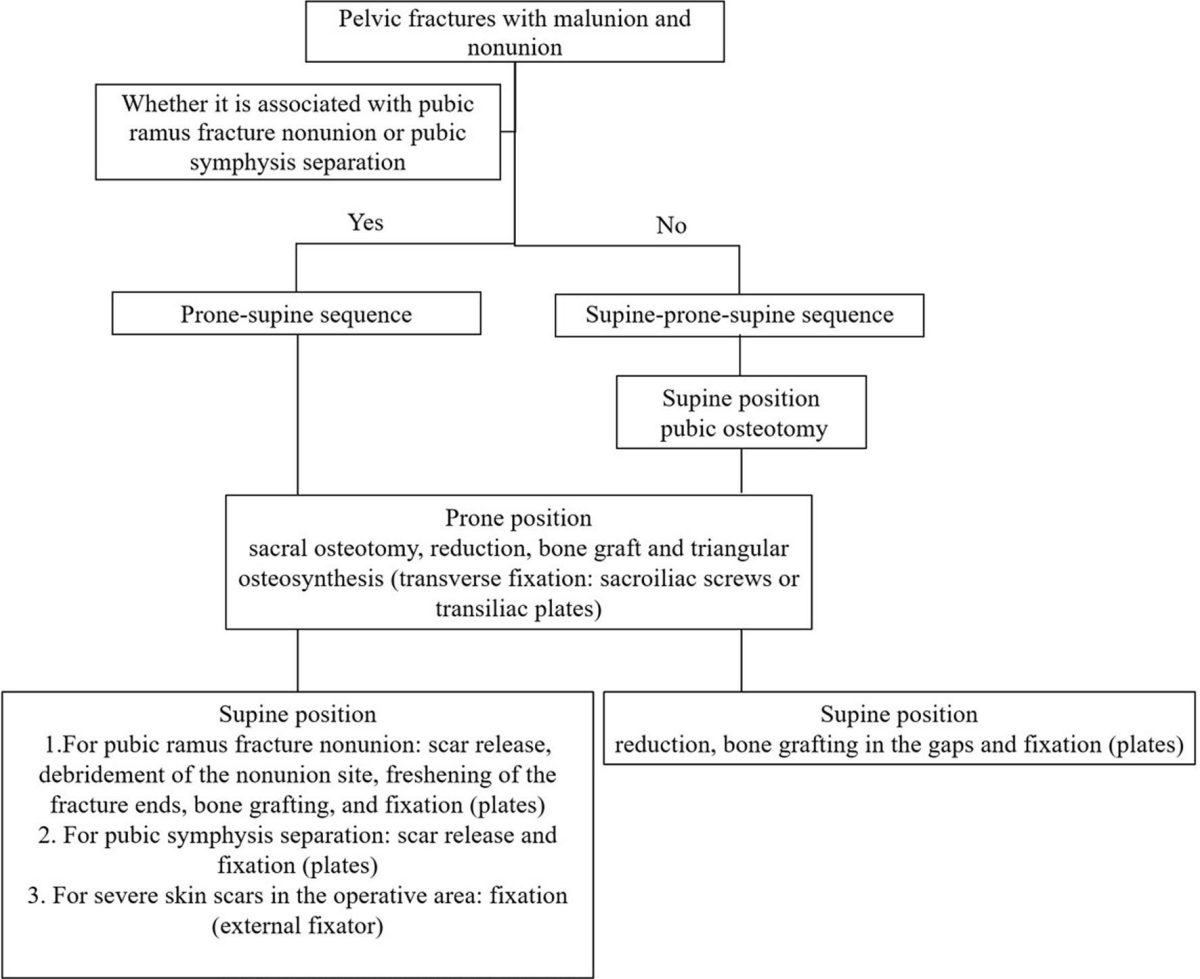
The figure of key surgical procedures

Pelvic malunion and nonunion require multistage surgery (Fig. 1). The sequence, either supine–prone–supine or prone–supine, was individualized in each case. In the supine–prone–supine sequence, after performing osteotomy of the superior and inferior pubic rami in the anterior pelvic ring in the supine position, the anterior surgical incision was closed temporarily. Subsequently, the patient was turned to the prone position, and sacral osteotomy, reduction, and internal fixation were performed, followed by closure of the incision. Then, turning the patient back to the supine position, the anterior surgical incision was reopened. After reduction and fixation of the pubic rami, the surgical incision was sutured. In the prone–supine sequence, after performing the sacral osteotomy, reduction, and fixation in the prone position, the incision was sutured; then, turning the patient to the supine position, anterior scar release, debridement of the nonunion site, freshening of the fracture ends, and bone grafting in the gaps were performed. After the anterior pelvic ring was reduced and fixed, the anterior surgical incision was closed.

**Fig. 2:**
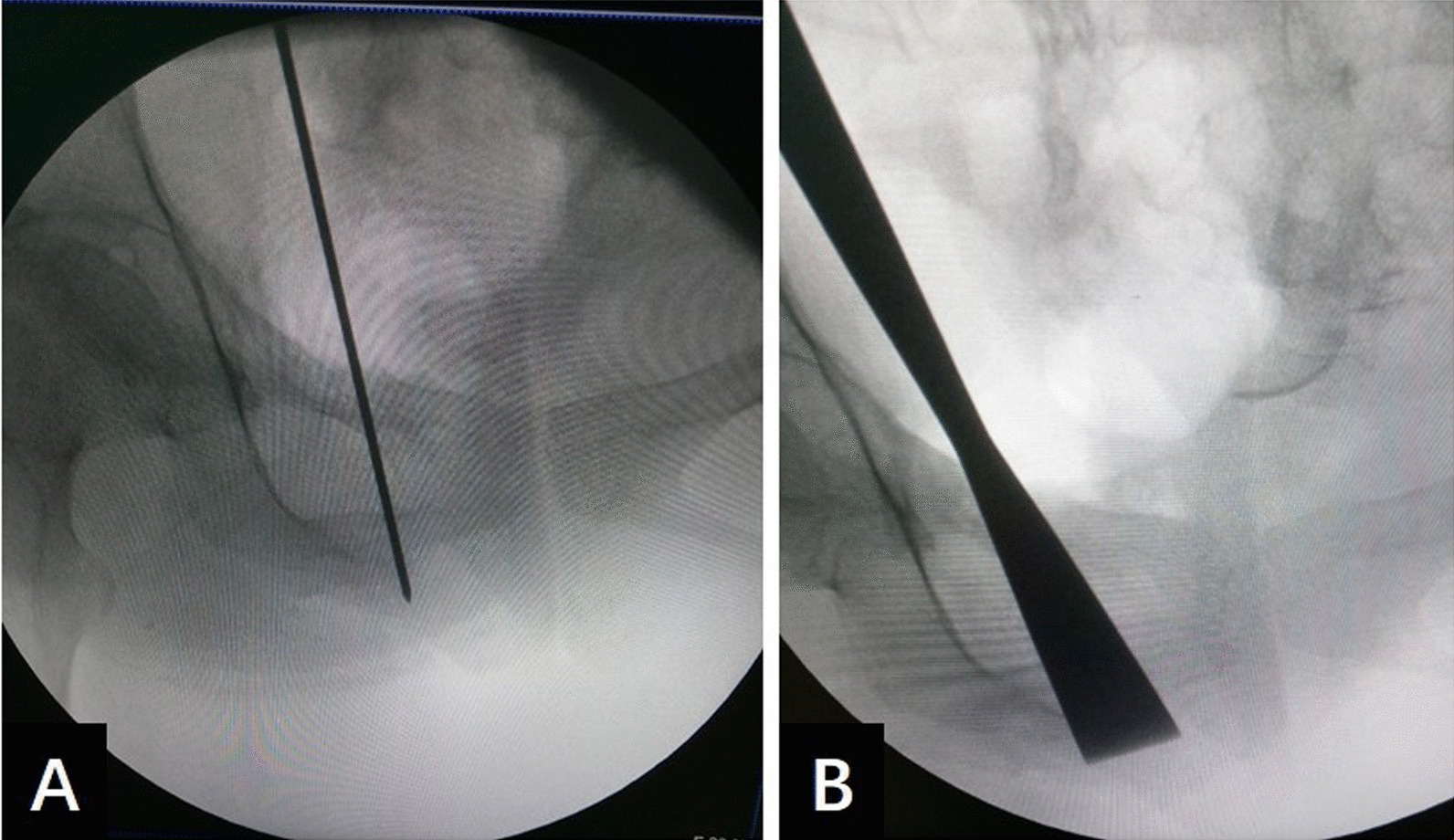
**a** Anteroposterior (AP) X-ray showing localization of the osteotomy line of the superior and inferior pubic rami with 2.0 K-wires. **b** Bone cut using an osteotome along the osteotomy line

The surgical approach for the anterior pelvic ring was the Stoppa incision. A midline incision was made 2 cm above the pubic tubercle to expose the pubic rami. The obturator fascia was dissected, and the obturator nerves and vessels were exposed. A 2.0-mm K-wire was used as the guide needle, which was inserted from the superior pubic ramus to the inferior pubic ramus, medial to the obturator neurovascular bundle. After determining the position of the guide needle under fluoroscopy, the superior and inferior pubic rami were cut along the guide needle (Fig. 2a, b). Complete disconnection of the superior and inferior pubic rami was confirmed with fluoroscopy; then, the surgical incision was closed temporarily.

**Fig. 3:**
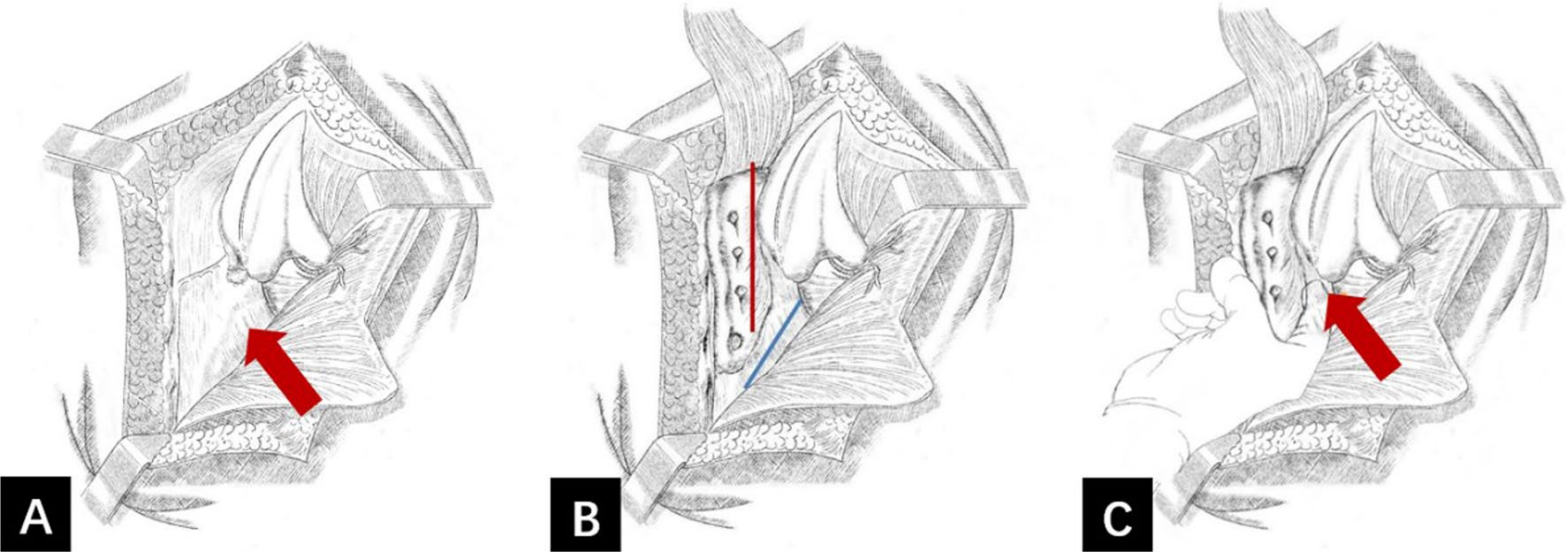
Schematic diagram for sacral osteotomy of the posterior pelvic ring. **a** Sacrotuberous ligament (red arrow). **b** Location of the sacral osteotomy (red line) and incised sacrotuberous ligament (blue line). **c** Schematic of the surgeon crossing the greater sciatic notch with the index finger to probe the anterior surface of the sacrum (red arrow)

**Fig. 4:**
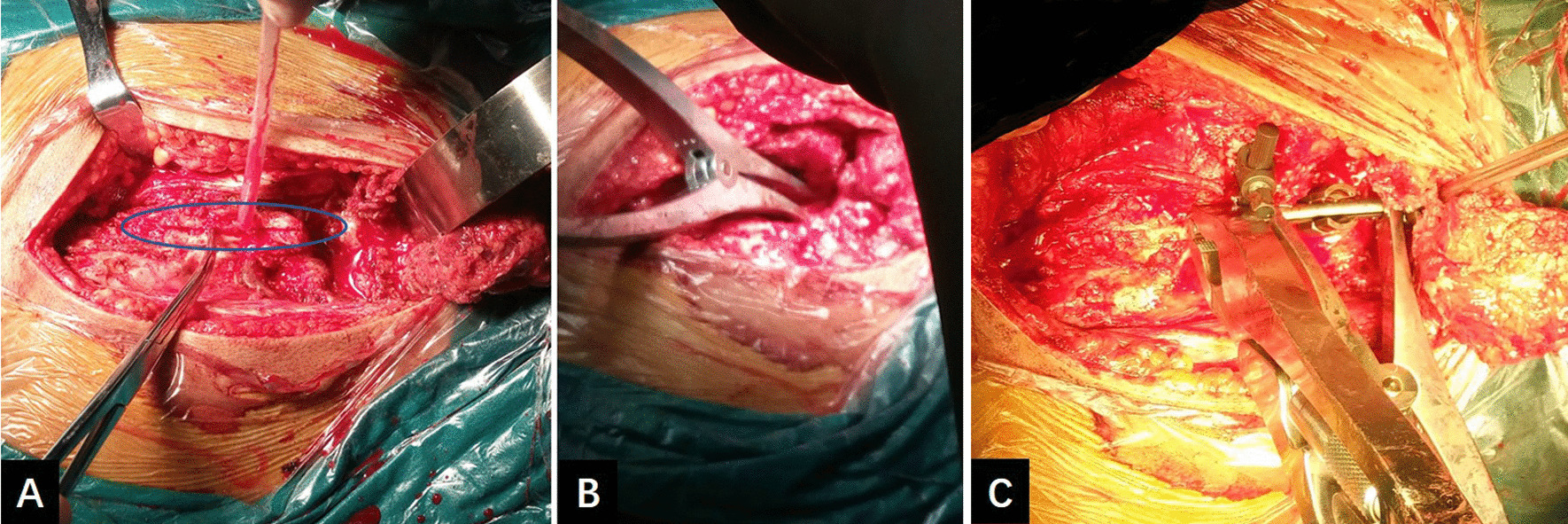
**a** Sacral osteotomy line (blue circle), located lateral to the sacral foramen. **b** Vertebral plate retractor used to separate the bone to confirm complete cutting and disconnection of the sacrum. **c** Distraction performed between the pedicle screws (L4 and L5) and the iliac screw for unilateral vertical pelvic displacement

Sacral osteotomy of the posterior pelvic ring was performed in the prone position. A longitudinal incision was made at a position 2 cm lateral to the L4-S3 spinous processes. The lumbodorsal fascia was dissected medially along the medial iliac crest and lateral margin of the sacrum; the entire sacral lamina, ala sacralis, and sacral foramen were exposed after lifting the sacrospinous muscle. Continuing dissection superiorly, the L4 and L5 vertebral plates, facets, and transverse processes were exposed. The sacrotuberous ligaments were cut along the sacral margin, across which the surgeon could use the index finger to sense the hard bone cortex on the anterior surface of the sacrum. For patients with a fracture history of more than one year, both the sacrotuberous ligament and the sacrospinous ligament attached to the sacrum were cut to reduce the superior unilateral pelvic migration. We chose the lateral side of the sacral foramen for sacral osteotomy (Fig. 3a–c). The osteotomy line was first made by drilling with a 2.0-mm K-wire; then, the osteotomy was performed, and a vertebral plate retractor was used to separate the broken bone to ensure complete cutting and disconnection of the sacrum (Fig. 4a, b). During the osteotomy, the surgeon could sense the hard bone cortex on the anterior surface of the sacrum with an osteotome as it approached the anterior cortex of the sacrum. While the osteotome was passed through the anterior cortex of the sacrum, the surgeon touched the sacroiliac joint and anterior surface of the sacrum anteriorly with his fingers across the greater sciatic notch to ensure that the osteotome did not penetrate 2–3 mm beyond the anterior surface of the sacrum, thereby preventing injury of the presacral vessels and nerves.

After reduction of the posterior pelvic ring, internal fixation was performed. The surgical instruments were the CDH™ LEGACY closed multiaxial screw sacroiliac internal fixation system (WEGO, Inc., Shandong, China), which is characterized by a connection module between the iliac screw and the connecting rod. The connection module allows the connecting rod to match the lumbosacral anatomical shape perfectly, simply by shaping on the sagittal plane and without multiplanar shaping. Pedicle screws were implanted in the L4 and L5 pedicles, and iliac screws were implanted in the posterior superior iliac spine. The iliac screw size was typically 7.5 × 80 mm. It was important that a bone window be made in the ilium with a rongeur to allow deeper seating of the iliac screw head, preventing prominence of the screw head under the soft tissues. Unilateral vertical pelvic displacement was reduced by longitudinal distraction between the L4 pedicle screw and the connecting module, and unilateral posterior pelvic displacement was reduced via the pulling action created by turning the L5 pedicle screw cap. After reduction of the anteroposterior displacement, the locking between the connecting module and the connecting rod was loosened. Further distraction reduction was performed between the component formed by the L4 and L5 pedicle screws together and the iliac screw, thereby preventing fixation failure due to the loosening of individual pedicle screws (Fig. 4c). After reduction was complete, the connecting module and connecting rod were relocked. Intraoperative AP, inlet, and outlet X-ray images were used to confirm the reduction effect. Transverse fixation was performed with sacroiliac screws or transiliac plates. Finally, returning the patient to the supine position, fixation of the pubic rami was performed.

**Fig. 5:**
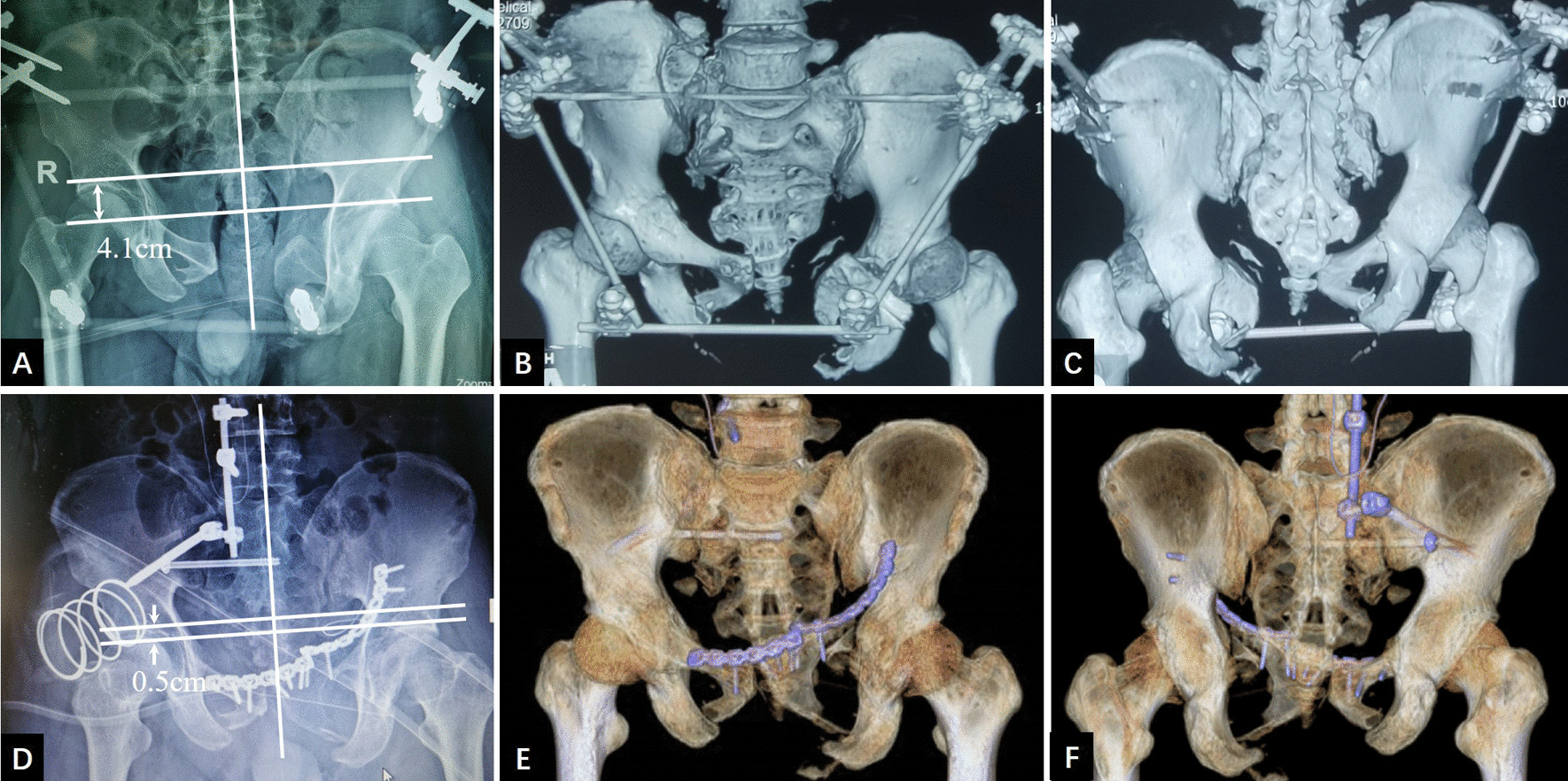
A 32-year-old male patient treated with the prone–supine sequence. **a** Preoperative AP X-ray showing significant vertical displacement (4.1 cm) of the right hemipelvis. **b, c** Three-dimensional CT showing separation of the anterior pelvic ring at the pubic symphysis and malunion of the posterior pelvic ring. **d–f** Postoperative AP X-ray and 3D-CT showing that a residual vertical displacement of 0.5 cm

For patients with pubic ramus fracture nonunion or pubic symphysis separation, the prone–supine sequence was used (Fig. 5). Autologous bone graft was applied at all osteotomy sites and freshened fracture ends.

### Postoperative management

Postoperative management included the use of prophylactic antibiotics and analgesic medication as well as prophylaxis for deep vein thrombosis. The surgical incision was drained for 48–72 h. Patients with neurological symptoms were given trophic nerve therapy. Patients were encouraged to perform active and passive exercises of the lower limbs in bed from day 1 after surgery. Nonweight-bearing activity was started 4 weeks after surgery. Full weight-bearing walking was allowed after the fracture healing 3 months later.

## Results

Among the nine patients, there were five males and four females, with an average age of 30.7 years. According to the AO/OTA classification of the initial fractures, there were eight cases of type C1.3 and one case of type C3.3 (the left sacral fracture showed no displacement, while the right sacral fracture showed vertical displacement and nonunion) fractures. According to the Denis classification, there were one case of Denis I and eight cases of Denis II fractures. The time from injury to surgery ranged from 3 months to 5 years, with an average of 12.8 months. The detailed basic data of these patients are presented in Table 1.

**Table 1:** Basic data of patients

Case no.	Age (years)	Gender	Cause	AO/OTA classification	Denis classification	Preoperative neurological deficit	Associated lesions	Delay to surgery
1	29	F	Fall	C1.3	II		Thoracic injury, abdominal injury	2 years
2	44	M	Motor vehicle accidents	C1.3	II		Right femoral fracture	5 years
3	32	M	Motor vehicle accidents	C1.3	II		Cerebral injury, right humerus fracture	3 months
4	19	F	Fall	C1.3	II	Yes	Right femur fracture	8 months
5	45	F	Motor vehicle accidents	C1.3	I		Abdominal injury	3.5 months
6	29	F	Motor vehicle accidents	C1.3	II		Abdominal injury, left ulnar fracture	3.5 months
7	31	M	Motor vehicle accidents	C1.3	II		Cerebral injury	4 months
8	14	M	Fall	C1.3	II		Abdominal injury	3 months
9	33	M	Motor vehicle accidents	C3.3	II		Abdominal injury, third-degree burn wound in the left inguinal region	6 months

F, female; M, male

The operative duration of 9 patients ranged from 3–7 h, with an average of 4.5 h. The intraoperative blood loss ranged from 1300–6100 mL, with an average of 2978 mL. Postoperative AP X-ray examinations showed that the correction of vertical displacement ranged from 1.7–3.9 cm (average, 3.2 cm). In one patient, the postoperative residual displacement was 1.5 cm (the preoperative vertical displacement was 3.2 cm), while in the other eight patients, the residual postoperative displacement ranged from 0.1–0.7 cm. These results are presented in Table 2.

**Table 2:** Operative data

Case NO	Postural sequence	Transverse fixation of the posterior pelvic ring	fixation of the anterior pelvic ring	Operative duration	Intraoperative blood loss	Cranial displacement	Correct displacement	Remarks
1	Supine–prone–supine	Transiliac plate	Plate	5 h	3600 mL	4.5 cm	3.8 cm	
2	Supine–prone–supine	Transiliac plate	Plate	5.5 h	3800 mL	3.8 cm	3.2 cm	Surgery in two stages^*^
3	Prone–supine	Sacroiliac screw	Plate	3.5 h	2600 mL	4.1 cm	3.6 cm	
4	Supine–prone–supine	Transiliac plate	Plate	7 h	6100 mL	3.3 cm	2.8 cm	
5	Supine–prone–supine	Transiliac plate	Plate	5 h	3300 mL	3.9 cm	3.4 cm	
6	Prone–supine	Transiliac plate	Plate	4 h	1800 mL	3.0 cm	2.7 cm	
7	Supine–prone–supine	Sacroiliac screw	Plate	4.5 h	2200 mL	3.2 cm	1.7 cm	
8	Prone–supine	Transiliac plate	Plate	3 h	2100 mL	4.3 cm	3.7 cm	
9	Prone–supine	Sacroiliac screw	external fixator	3 h	1300 mL	4.5 cm	3.9 cm	

*Surgery in two stages: In this case, the intraoperative reduction was difficult after osteotomy; in the first stage of surgery, anterior and posterior ring osteotomy was performed; in the two-stage operation, posterior and anterior ring fixation was performed

**Fig. 6:**
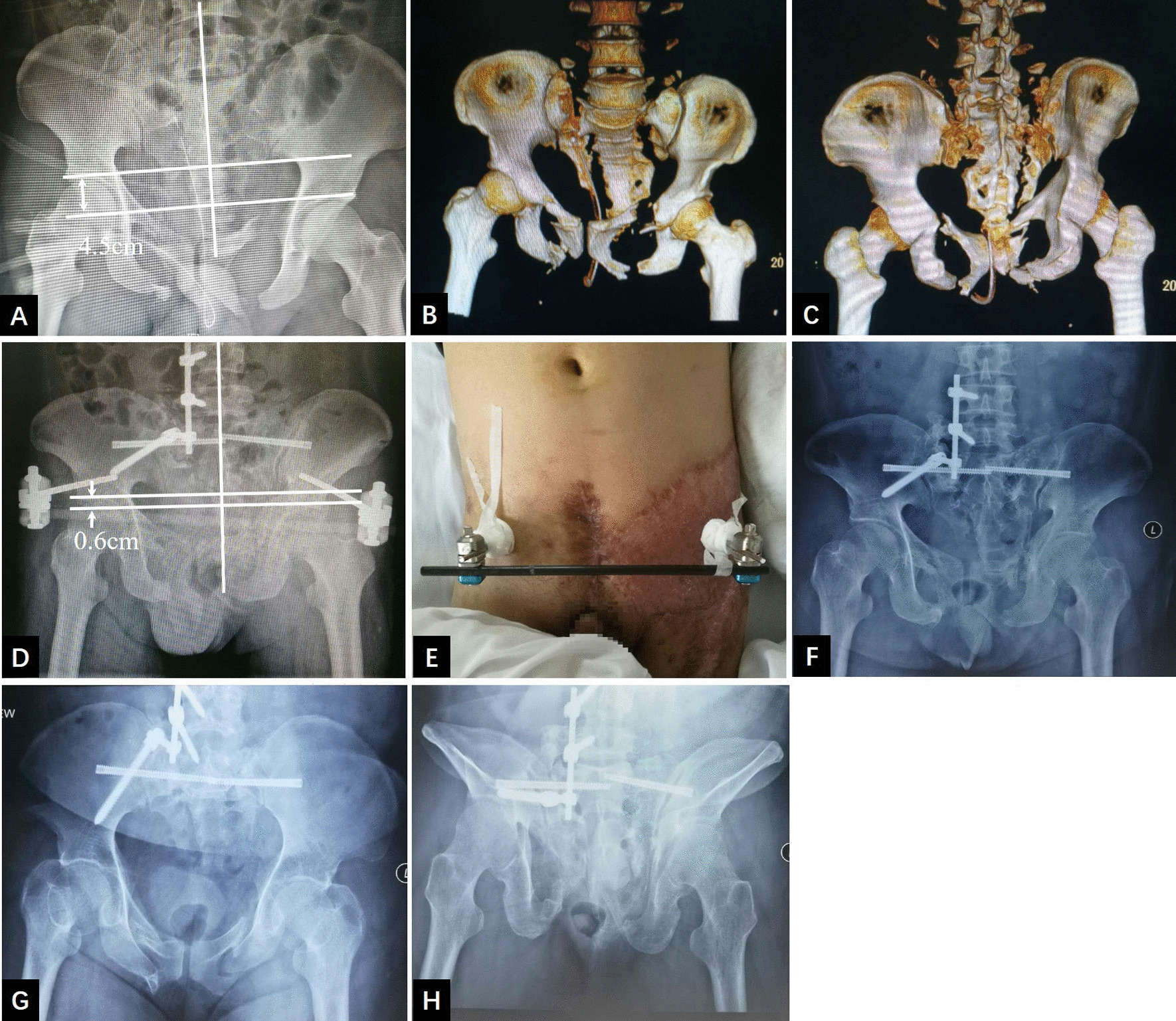
A 44-year-old male patient with a type C3.3 pelvic fracture. **a–c** Preoperative AP X-ray and three-dimensional CT showing significant vertical displacement (4.5 cm) of the right hemipelvis. **d** Osteotomy of the right sacrum, followed by lumbopelvic distraction osteosynthesis, bone grafting, and bilateral sacroiliac screw fixation, resulting in a residual unilateral vertical pelvic displacement of 0.6 cm. **e** Nonunion of the pubic rami combined with severe third-degree burn scars in the left inguinal region necessitating, external fixation of the anterior pelvic ring. **f–h** AP, inlet, and outlet X-ray at the six-month follow-up showing no displacement of the fracture (the external fixator of the anterior pelvic ring was removed 3 months after surgery)

**Fig. 7:**
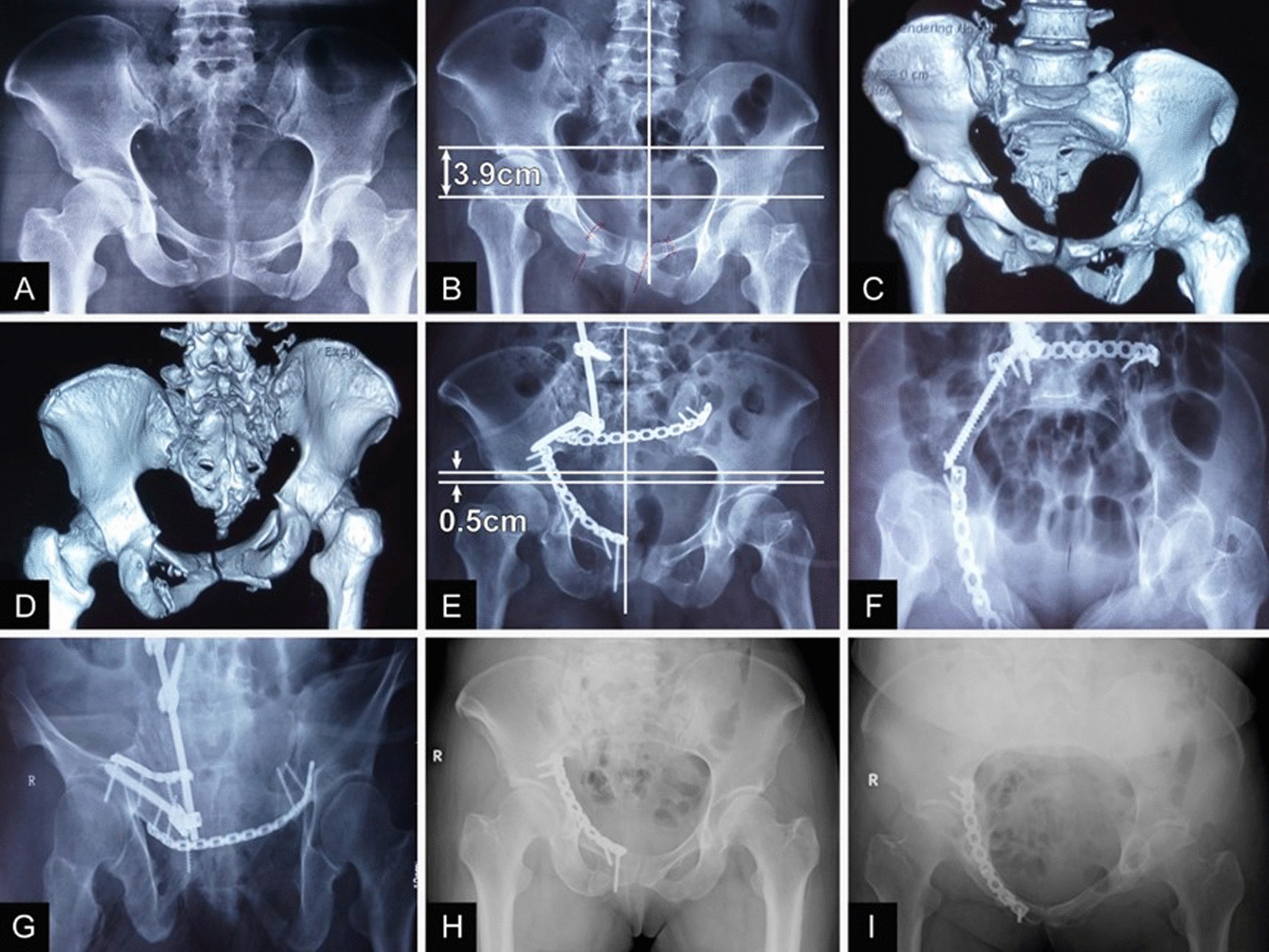
A 45-year-old female patient. **a** AP X-ray at the time of injury showing the pelvic ring without obvious cranial displacement. **b** AP X-ray showing 3.9 cm of unilateral cranial pelvic displacement after 1 month of conservative treatment. **c, d** Three-dimensional CT showing sacral malunion at 3 months after the failure of reduction of femoral skeletal traction. **e** Postoperative AP X-ray showing a residual displacement of 0.5 cm after reduction. **f, g** Inlet and outlet X-ray images showing reduced hemipelvic displacement. **h, i** Pelvic radiographs acquired 15 months after the operation showing healing of the fracture and removal of the instrumentation for internal posterior pelvic ring fixation

Five patients were treated with the supine–prone–supine surgical sequence, and four were treated with the prone–supine sequence. One patient with a vertical displacement of 4.3 cm before surgery exhibited plantar numbness after surgery but without dyskinesia, which recovered after one month of neurotrophic therapy. Transverse fixation of the posterior ring was performed with sacroiliac screws (Figs. 5d, 6d) in three cases and transiliac plates (Fig. 7e) in six cases. The anterior pelvic ring was fixed with plates (Fig. 5d) in eight cases and an external fixator (Fig. 6e) at the upper edge of the acetabulum in one case. This case involved a third-degree burn in the left inguinal region with severe skin scars; this patient was treated with an external fixator at the upper edge of the acetabulum with no soft tissue release of the anterior pelvic ring (Fig. 6). Eight patients were treated with primary fixation. In one case of pelvic malunion for 5 years, the intraoperative reduction was difficult; after anterior and posterior ring osteotomy, the surgical incision was temporarily closed. One week of augmented combined traction of the femoral/pelvic area was applied, followed by posterior and anterior ring fixation in the second-stage operation. For the two patients with fracture lines involving sacral zone II and nonunion, unilateral laminectomy was performed first, followed by careful nerve root dissection and scar and sclerotic bone removal from the fracture ends. Subsequently, the osteotomy was performed laterally in the sacral foramen. In one patient with symptoms of sacral plexus injury preoperatively, the S1-3 nerve roots ruptured during the operation.

The mean postoperative follow-up time of the 9 cases was 12 months (6–36 months). Eight patients had no significant postoperative claudication, and one patient had mild claudication. One patient had a preoperative sacral plexus injury without recovery of ankle motor function, and ankle arthrodesis was performed one year later. One patient showed postoperative nonunion of the pubic rami that was not managed as it was asymptomatic, and the remaining eight patients showed healing at the osteotomy sites on X-ray and CT examinations. No iatrogenic nerve lesions were observed in any of the patients. No patients showed any loss of reduction, and no case of implant breakage or loosening was observed. At the last follow-up, none of the patients had pain or movement-related pain at the osteotomy sites. The Majeed score increased from an average of 54 points (30–84 points) preoperatively to 87 points (72–94 points) postoperatively. The VAS score for pain decreased from an average of 6.0 points (4–8 points) preoperatively to 1.2 points (0–3 points) postoperatively (Table 3).

**Table 3:** Postoperative data

Case no.	VAS		Majeed score		Follow-up time	Remarks
	Preoperative	Postoperative	Preoperative	Postoperative		
1	5	1	84	94	6 months	
2	4	1	76	88	1 year	Right femoral nonunion with shortening of 2 cm
3	7	2	41	91	6 months	
4	6	0	76	91	18 months	Operated at the local hospital, 2 screws on anterior ring plate were penetrated the acetabulum
5	8	1	34	93	1 year	
6	7	0	30	89	3 years	
7	6	3	44	72	6 months	Operated at the local hospital, failed to correct the upward displacement
8	7	2	33^*^	73^*^	6 months	6 cm × 11 cm bedsore on sacrococcygeal region
9	4	1	67	92	6 months	

VAS, pain visual analog scale score

*This 14-year-old boy was underage, and the sexual intercourse of items was calculated as a full score of 4

## Discussion

With the promotion and popularization of early emergency treatment and reconstruction techniques for pelvic fractures, the success rate concerning the early management of pelvic fractures has greatly improved, and most displaced unstable pelvic fractures can be reduced and fixed early [10]. However, some severe pelvic fractures do not qualify for early reconstruction due to the need for early rescue efforts for life-threatening massive bleeding and associated injuries [3, 11]. On the one hand, some doctors lack an understanding of the degree of pelvic instability and are unable to apply correct and adequate vector forces to reduce and stabilize the fractures. On the other hand, primary hospitals are limited by the available hardware and techniques, resulting in the occurrence of malunion or nonunion. In our study, one patient with a type C1.3 pelvic fracture showed no obvious displacement after injury and was given conservative treatment with bed rest due to insufficient understanding of the instability of the pelvic fracture by the surgeon. One month later, the AP X-ray examination revealed 3.9 cm of unilateral vertical pelvic displacement. The patient underwent reduction using femoral skeletal traction, which failed; 3.5 months later, the patient presented to our hospital with obvious claudication. Then, at our hospital anterior and posterior pelvic ring osteotomy, reduction and stable internal fixation were performed, and follow-up at 8 and 15 months after surgery showed no loss of reduction (Fig. 7). Another patient had pubic ramus fractures of the anterior pelvic ring and sacral zone II fractures of the posterior pelvic ring after injury, with significant vertical displacement and symptoms of sacral plexus nerve injury. Because the surgeon was unable to apply correct and adequate vector forces to reduce and stabilize the fractures and was operating under technical limitations, only the pubic rami of the anterior ring were fixed in the initial treatment; the vertical displacement of the posterior ring was not reduced, and the sacrum was not fixed. Furthermore, two screws of the anterior ring plate were mistakenly inserted into the acetabulum, resulting in claudication and hip and lumbosacral pain in the patient for up to 18 months postoperatively.

In recent years, due to the clinical application of the pelvic reduction frame, some pelvic fractures can be treated with closed reduction and minimally invasive fixation in the early stage, with better results and a lower incidence of pelvic malunion or nonunion [12, 13]. However, the application of this new technique has not been popularized, and some complex pelvic fractures are difficult to correct with reduction frames. Besides, the difficulty of open surgical techniques and the intolerance of patients' early physiological conditions to open surgery discourage surgeons. Thus, pelvic malunion and nonunion still occur.

The treatment of pelvic malunion and nonunion is challenging for surgeons because of significant intraoperative difficulties and postoperative uncertainty. Few treated cases have been reported in the literature [3]. Therefore, there are no standard treatment strategies for pelvic malunion and nonunion; however, the ultimate treatment goal remains the same: to restore the integrity and symmetry of the pelvic ring, carefully manage soft tissue, facilitate rapid postoperative recovery and early rehabilitation, and achieve long-term functionality of the hip joint.

Significant vertical displacement deformity is a common reason for patients with pelvic malunion or nonunion presenting to the hospital. It is very difficult to correct such deformity. On the one hand, the pelvic ring fracture line has formed a callus, which needs to be converted into a fresh fracture by osteotomy. On the other hand, the severe contracture of soft tissue requires extensive release. Moreover, numerous nerves, blood vessels, and other vital structures surrounding the pelvic region further increase the difficulty of surgery and the risk of treatment.

The surgical treatment of pelvic malunion and nonunion depends on the clinical symptoms, the degree of vertical pelvic displacement, and the requirements of the patients. It is important to ascertain whether the symptoms are related to pelvic malunion or nonunion rather than to other clinical conditions; pain from low back mechanics or an old neurological injury and dysesthetic pain of neurogenic origin must be excluded [6]. Pain at the site of pelvic fractures is often caused by nonunion; however, in some cases, this pain is caused by the nerve injury that occurs during the initial pelvic fracture, which is often difficult to eliminate [14]. We must inform patients that pelvic pain not associated with nonunion or instability is not corrected with this type of surgery [6]. Furthermore, reconstructive surgery involves the risk of many complications, including nerve injury (5.3%), symptomatic venous thrombosis (5.0%), pulmonary embolism (1.9%), and deep wound infection (1.6%) [14]. A hemipelvic displacement of > 1 cm or rotation of 15° to 20° may not always be clinically symptomatic, but the combination of these conditions is much more likely to be clinically symptomatic and may represent malunion [15]. Pelvic fractures with significant displacement often cause gait instability and claudication. All patients in our study had sacral fractures of the posterior pelvic ring, vertical displacement > 2 cm, and different degrees of gait abnormalities. The patients had a strong desire for surgery to improve existing symptoms and restore lower limb length symmetry. The authors deem it acceptable for a hemipelvic vertical displacement of ≤ 1 cm and/or rotation of < 15°–20° displacement. However, some studies have suggested that surgical correction is indicated in cases of rotational defects greater than 10°, leg length discrepancies greater than 5 mm, and a lack of reduction or imperfect facing of sacroiliac articular surfaces [14].

In the process of reconstruction, the malunion site requires osteotomy, and the scar contracture site requires release. Methods of posterior pelvic ring osteotomy include the iliac and sacral osteotomy, both of which are able to correct pelvic obliquity and restore lower limb length symmetry [14, 16]. The authors believe that iliac osteotomy is mainly applied to type B pelvic fractures involving the ilium and pelvic obliquity, while sacral osteotomy is mainly used for longitudinally displaced deformities originating from sacral fractures. Given that this group of patients had sacral fractures (C1.3 or C3.3) of the posterior pelvic ring and significant unilateral vertical pelvic displacement, the sacral osteotomy method was better for restoring the shape of the pelvis.

Posterior pelvic ring fixation methods include transverse osteosynthesis, vertical osteosynthesis, and triangular osteosynthesis. Transverse osteosynthesis usually involves sacroiliac screws, sacral rods, and transiliac and transsacral plates, which exhibit poor shear resistance [2]. Vertical osteosynthesis can be achieved by LPDO. When LPDO is combined with transverse osteosynthesis, triangular osteosynthesis is achieved, which provides stability against longitudinal displacement and rotation and promotes early postoperative weight-bearing and rehabilitation [2, 17]. In addition, the longitudinal distraction effect between the lumbar spine and pelvis in triangular osteosynthesis can correct unilateral vertical pelvic displacement directly. Triangular osteosynthesis was applied in all patients in our study.

The small number of cases in this study precludes statistical analysis and presents some limitations. In addition, the data used in this study were not sufficiently accurate for measuring the rotational deformity of the pelvis, which will be our main research direction in the future.

## Conclusion

Sacral osteotomy combined with triangular osteosynthesis for vertically displaced pelvic fractures with malunion and nonunion significantly improves pain, deformity, and claudication and can be used as an effective treatment option for severe vertically displaced pelvic deformity following sacral fractures.

## Data Availability

Please contact author for data requests.

## Abbreviations

3D-CT: Three-dimensional computed tomography
LPDO: Lumbopelvic distraction osteosynthesis
VAS: Visual analogue scale
AP: Anteroposterior
